# The Changing Face of Hepatitis Delta Virus Associated Hepatocellular Carcinoma

**DOI:** 10.3390/cancers16223723

**Published:** 2024-11-05

**Authors:** Mariana Ferreira Cardoso, Mariana Verdelho Machado

**Affiliations:** 1 Gastroenterology Department, Hospital Prof. Doutor Fernando Fonseca, 2720-276 Amadora, Portugal; marianafcardoso@gmail.com; 2 Clínica Universitária de Gastrenterologia, Faculdade de Medicina, Universidade de Lisboa, 1649-028 Lisbon, Portugal; 3 Gastroenterology Department, Hospital de Vila Franca de Xira, 2600-009 Vila Franca de Xira, Portugal

**Keywords:** hepatitis delta virus, hepatocellular carcinoma, epidemiology, pathogenesis

## Abstract

Hepatitis delta virus (HDV) infection requires the presence of hepatitis B virus (HBV), and chronic HBV–HDV coinfection is considered the most severe form of viral hepatitis. When compared with HBV mono-infection, HBV–HDV coinfection is associated with higher rates of liver cirrhosis and hepatocellular carcinoma (HCC). In this review, we aim to elucidate the complex relationship between HDV infection and the development of HCC. The exact mechanisms underlying the carcinogenic potential of HDV remain to be fully elucidated. Evidence suggests that HDV has both indirect and direct oncogenic effects. Indirect effects promote accelerated progression to liver cirrhosis, which results in a different tumor microenvironment. Direct oncogenic effects are suggested by a distinct molecular signature. The recent epidemiological data regarding HBV–HDV coinfection should make us reconsider the HCC screening strategy, with special focus in younger non-cirrhotic patients. Finally, treating HCC in patients with chronic HDV poses unique challenges due to the complex interplay between HBV and HDV and the severity of liver disease. An in-depth understanding of the epidemiology and pathophysiology of HDV infection and carcinogenesis is essential to improve disease management in this high-risk population.

## Simple Summary

Hepatitis B virus (HBV)—hepatitis delta virus (HDV) coinfection accounts for an estimated 8–10% of cases of hepatocellular carcinoma (HCC) worldwide. Recent meta-analyses suggest that HDV increases the risk of HCC associated with HBV, but its pathogenic role remains poorly elucidated. HCC related to HBV–HDV coinfection tends to be diagnosed by surveillance but in the background of severe liver dysfunction, which has therapeutic implications, with a high rate of liver transplantation, and high mortality in patients not eligible for liver transplantation.

## 1. Introduction

Hepatocellular carcinoma (HCC) is a major health threat worldwide, corresponding to the sixth incident cancer and the third cause of cancer mortality [1]. The higher position in the ranking of mortality compared to the ranking of incidence reflects its dismal prognosis, with less than 1 in 5 patients surviving beyond 5 years after the diagnosis [2]. The burden of HCC presents geographical disparities, with Asia accounting for 70% of HCC, Europe 10%, and North America only 5% [1]. These differences align with the unevenness in the prevalence of hepatitis virus infections, particularly hepatitis B virus (HBV), which accounts for over 40% of HCC worldwide [3]. Importantly, up to 20% of HBV-associated HCC develop in the setting of HBV–hepatitis D virus (HDV) coinfection [4].

HDV was first described in 1977, in Italy, by Mario Rizzetto [5]. It is a small defective RNA virus that can only infect and persist in hepatocytes in association with HBV, taking advantage of HBsAg (HBV surface antigen) to produce its envelope [6]. HDV coinfects 4.5–13% of HBV-infected patients [4,7,8], and it is estimated that more than 50 million people are chronically infected worldwide [8].

HDV does not have a direct cytopathic effect, nor does its genome integrate with the host genome. However, HBV–HDV coinfection is believed to be the most aggressive form of chronic viral hepatitis, driven by immune-mediated injury [9], with an up to four-fold higher risk of accelerated progression to liver cirrhosis, hepatocellular carcinoma, end-stage liver disease, and mortality [8,10,11,12,13]. On average, in a patient with chronic HBV–HDV infection, it takes 5 years to progress to liver cirrhosis and 10 years to develop HCC [8]. This represents an accelerated course compared to other forms of chronic viral hepatitis such as HBV and HCV mono-infection that takes 20 and 30 years [14,15] to develop cirrhosis or HCC, respectively.

The field of HDV infection has recently gained renewed awareness following advances in its medical treatment, with recent international guidelines recommending universal screening of HBV patients for anti-HDV antibodies.

In this review, we critically summarize the latest data in the epidemiology, pathogenesis and clinical relevance of HBV–HDV coinfection-associated HCC.

## 2. Epidemiology of HDV-Associated Hepatocellular Carcinoma

The impact of HDV infection in the development of HCC has been debated for decades. The classical view on the matter was that HDV infection was associated with a severe and rapidly progressive liver disease, leading to liver transplantation or death from liver decompensation before the onset of HCC, which was regarded as a late complication of the disease. In fact, early reports from Italy, where the virus was originally described in the 1970s [5], showed a concerning scenario, with fast progression to cirrhosis and decompensation [16,17]. A later report from Milan, from 15 years ago, although less alarming than the former, still stated that, differently from HBV and HCV, it was clinical decompensation, rather than HCC, that dominated the clinical course of HDV patients [18]. Indeed, at the diagnosis of HDV chronic hepatitis, one to two thirds of the patients have established cirrhosis, and more than half will die of liver disease in the first 10 years after the diagnosis [19]. More recently, however, in Europe, where immigrants contribute to more than half the HDV burden [7], less virulent strains have been identified in younger immigrants from African countries, as compared to domestic ageing survivors of infection [20,21], resulting in more than half of the patients presenting an indolent course [22,23,24,25], allowing for hepatocarcinogenesis to occur.

Wrapping up, current evidence suggests that, when compared to HBV mono-infection, HBV–HDV coinfection is associated with faster progression to cirrhosis and decompensation [21,26,27]. A recent meta-analysis, which included mostly studies published after 2020, described a 70% increased risk of liver cirrhosis, 3-fold increased risk of HCC, 7-fold of liver transplantation, and almost 4-fold of liver-related mortality, in HBsAg carriers with active HDV infection, defined by detectable HDV RNA [28]. Even though HBV–HDV coinfection occurs in only around 5% of HBsAg carriers, one fifth of HBV-associated cirrhosis, as well as HCC cases, are attributable to HDV coinfection [4].

The first cohort study claiming HDV coinfection as a risk factor for HBV-associated HCC was the landmark study by Fattovich et al., published in 2000 [29]. In this prospective multicentric study, 200 Caucasian patients from Western Europe with untreated HBV infection and compensated cirrhosis, including 20% with anti-HDV positivity, were followed for a median of 6.6 years. After adjusting for confounders, including age, HCC risk in HBV–HDV coinfected patients was three-fold higher, compared to HBV mono-infected patients. Since that first study, however, published studies produced conflicting results [10,11,12,30,31,32,33].

In order to tackle this question, three systematic reviews with meta-analyses have been conducted to date [34,35,36]. All of them pointed in the same direction, showing a two-fold increased risk of HCC in HBV–HDV coinfected patients as compared to HBV mono-infected patients, when only prospective high-quality studies were included. Interestingly, in the subset of patients with cirrhosis, even though the tendency to higher risk remained, it did not reach statistical significance. This lack of statistical difference in the subgroup of cirrhosis, could be explained by the hypothesis that HDV-enhanced hepatocarcinogenesis is dependent on its enhanced progression to cirrhosis and that, after the cirrhotic state is reached, the association of cirrhosis and carcinogenesis is so strong that it blunts the potential oncogenic effect of HDV. These data suggest that most, if not all, of the increased carcinogenesis attributable to HDV occurs in the pre-cirrhotic stage. Unfortunately, there are very little data specifically concerning the non-cirrhotic population. A US nationwide cross-sectional study in non-cirrhotic patients showed a 50% increased risk of HCC in HBV–HDV coinfected as compared to HBV mono-infected patients [37]. These figures are lower than the two-fold increase reported for the whole spectrum of HDV-related disease (including cirrhotic and non-cirrhotic patients), which could potentially be explained by the use of strict criteria for excluding cirrhosis in the US study. More studies are needed to ascertain the risk of HDV-associated HCC in the pre-cirrhotic stage.

In the subgroup of patients living with human immunodeficiency virus (HIV), HBV–HDV coinfection confers an even higher risk of HCC, of around three-fold, compared to HBV mono-infection, which could be explained by an impaired immune surveillance caused by HIV, that can boost the oncogenic process [34]. Another noteworthy finding from the three mentioned meta-analyses was a stronger increase in HCC risk in studies performed after 2010, which the authors speculated could be explained by improvements in the treatment of cirrhosis and its complications throughout the years, resulting in increased survival. This, in turn, would have allowed for carcinogenesis to occur, in contrast to the more historical perception of decompensation-related mortality outlined above [16,17,18]. A stronger association between HDV and HCC after exclusion of HCV-infected patients was described and attributed either to a more robust design of these studies or to complex viral dominance patterns [34,36]. Globally, all three meta-analyses mention similar limitations, including study heterogeneity, large time-span, and lack of relevant data such as HBV treatment or HDV RNA status in most studies [34,35,36].

More recently, the Italian Liver Cancer (ITA.LI.CA) group published an analysis of their database, including more than 10,000 patients with HCC. The authors focused on a cohort of 695 patients with HBV infection, of which 107 (15.4%) had HDV coinfection [38]. Taking into account the estimated prevalence of HBV and HDV infections in Italy, these figures translated into a 1.8-fold greater risk of HCC in HBV–HDV coinfected patients [39,40], similarly to the findings of previous meta-analyses. This study did not compare the proportion of cirrhotic patients in both groups.

Lastly, active HDV replication was consistently found to be a risk factor for HCC development, resulting in a three-fold increase in the long-term risk [11,20,22,28]. However, once cirrhosis is established, HDV viremia loses its ability to predict the susceptibility to HCC [11], and HCC can occur even in the absence of active HDV infection, in patients with undetectable serum HDV RNA [31]. When analyzing the role of HDV replicative status in HCC and other liver-related outcomes, it should be noted, however, that the sensitivity of HDV RNA tests used in early studies was suboptimal [41]. This fact, combined with the intermittent nature of HDV viremia, advises caution when interpreting the results of those studies.

The main epidemiological studies concerning the impact of HDV in HCC development are detailed in Table 1. HBV mono-infected patients are used as reference, except when specified otherwise.

## 3. Mechanisms of HDV Promotion of Hepatocarcinogenesis

The role of HDV in hepatocarcinogenesis is yet to be fully unraveled. As explained above, natural history longitudinal studies strongly suggest that chronic coinfection with HBV–HDV is associated with a higher risk of developing HCC, as compared with chronic HBV mono-infection [29,30,42,44], which positively correlates with HDV viral load [20,28]. However, different meta-analyses failed to show an increased risk associated with coinfection once cirrhosis was established [34,35,36]. Indeed, liver cirrhosis is the main risk factor for HCC development, with more than 70% of HBV-associated HCC developing in patients with cirrhosis [45]. As such, the simplest explanation for a higher risk of HCC development in patients coinfected by HBV–HDV as compared to HBV mono-infection is the higher risk and accelerated progression to liver cirrhosis [10,12,13,18,20]. Indeed, HDV coinfection independently increases the production of profibrogenic factors such as transforming growth factor (TGF)-β, and enhances proinflammatory responses [46,47,48], explaining the worse prognosis of coinfection.

Even though accelerated progression to liver cirrhosis is probably the main mechanism of HDV-associated hepatocarcinogenesis, epidemiological and clinical studies suggest an independent HDV-mediated oncogenic effect. Indeed, as mentioned above, HDV seems to confer at least a 50% higher risk of HCC in the non-cirrhotic population [37]. This is even more relevant if we take into account that HBV oncogenic mechanisms (such as integration of viral DNA in the host genome and pleiotropic actions of HBsAg and HBx protein [49]) are proportional to HBV replication [50], which is frequently blunted by HDV [51], reinforcing the possible HDV oncogenic potential. Similarly, HBV–HDV coinfection most frequently associates with a negative HBeAg status [52]. HBeAg may promote hepatocarcinogenesis by evasion of immune response and by interfering with apoptosis [53]. The fact that most coinfected patients are HBeAg-negative, which might be associated with a lower HBV oncogenic potential, reinforces the concept a direct role of HDV in hepatocarcinogenesis.

Additionally, studies on gene expression profiling in liver tissue from patients with HCC found different molecular signatures in HBV–HDV coinfection-associated HCC from HBV mono-infection-associated HCC, suggesting different carcinogenic mechanisms [54,55,56]. The molecular signature of HDV-associated hepatocarcinogenesis conveys deregulation of the cell cycle, DNA replication and damage repair, and associated genomic instability [54,55].

HDV is not a direct carcinogen [57], nor does it appear to be associated with a direct cytopathic effect [58]. However, HDV may promote hepatocarcinogenesis through pleiotropic actions of HDV antigens, inducing oxidative stress, modulating different key cellular signaling pathways, as well as through epigenetic mechanisms.

HDV can induce oxidative stress by different mechanisms: (1) L-HDAg can induce the gene expression of NADPH oxidase 4 (Nox4 gene), an enzyme that produces reactive oxygen species (ROS) through the reduction of molecular oxygen [59]; (2) S-HDAg can downregulate glutathione S-transferase P1 (GSTP1) protein expression by specifically binding to GSTP1 mRNA [60]. Interestingly, GSTP1 is a tumor suppressor gene that has been linked to HCC, either by HCC-associated promoter hypermethylation [61,62], or by polymorphisms conferring higher risk of HCC development [63] and HCC lower survival [64].

HDV-induced oxidative stress disrupts mitochondrial homeostasis, ultimately culminating in signal transducer and activator of transcription-3 (STAT-3) and nuclear factor kappa B (NF-κB) activation [59].

STAT-3 is a known oncogene with critical roles in the regulation of the cell cycle, cell proliferation, apoptosis, and tumorigenesis [65]. In vitro studies in HDV hepatocarcinogenesis showed that STAT-3 pathway activation results in the upregulation of DNA methyltransferase beta (DNMT3b) [66], an enzyme that catalyzes de novo methylation of CpG nucleotides in promoter regions, leading to gene silencing. Remarkably, upregulation of DNMT3b has been described in HCC, conferring worse prognosis [67]. HDV-upregulated DNMT3b can silence tumor suppressor genes. It can also silence E2F1, a transcription factor that promotes expression of genes involved in S-phase entry, DNA synthesis and mitosis, as well as being involved in the regulation of DNA damage response and apoptosis [68]. The indirect HDV-upregulation of E2F1 seems to promote a G2/M cycle arrest, which might induce pressure on hepatocytes to acquire additional mutations to escape G2 [66].

An alternative mechanism for NF-κB HDV-induced overexpression is the L-HDAg-triggered degradation of the NF-κB inhibitor, IκB, mediated by tumor necrosis factor (TNF)-α [47]. NF-κB overexpression is a molecular trait of some HCC, and results in increased proliferation, decreased apoptosis, and a metastatic phenotype of tumor cells [69].

In vitro proteomic studies showed that S-HDAg induces degradation of the tumor suppressor p53, resulting in downregulation of 14-3-3δ. As 14-3-3δ binds to the phosphorylated CDK1-cyclin B complex, allowing its export into the cytoplasm, downregulation of 14-3-3δ results in dephosphorylation of the CDK1-cyclin B complex that remains in the nucleus, and hence can mediate premature induction of mitosis, increasing cell proliferation [70].

Another player in cell cycle regulation that has been linked to HDV-hepatocarcinogenesis is RBM5, a nuclear RNA binding protein known to induce cell cycle arrest and apoptosis by mediating preRNA splicing of multiple target genes such as p53 [71]. HCC has been linked to decreased expression of RBM5 [72]. HDV genomic RNA can directly interact with the splicing factor SF3B15S, leading to alternative splicing of several target transcripts, such as RBM5, with a consequent decrease in its protein levels [71].

HDV can also epigenetically increase the expression of oncogenes. For example, in vitro studies showed that both L-HDAg and S-HDAg can enhance the expression of the oncogene clusterin via histone 3 acetylation [73]. Clusterin overexpression has been associated with HCC [74], increasing the malignant cells survival potential [75] and migration/metastatic phenotype [76]. Other epigenetic mechanisms that are starting to be unraveled are the different expressions of long non-coding RNA (lncRNA) induced by different viruses. For example, HDV infection is associated with downregulation of Y3 lncRNA, an essential regulator of cell fate [77].

The TGF-β signaling pathway might also be involved in the oncogenic mechanisms of HDV. Indeed, isoprenylated L-HDAg can promote TGF-β and c-Jun pathway activation in a synergistic fashion with the HBV protein HBx [46]. TGF-β can promote hepatocarcinogenesis because, although it induces apoptosis in normal hepatocytes [78], it also increases proliferation [79] and self-renewal capacity of the hepatic progenitor cells [80]. Furthermore, malignant cells escape the proapoptotic effects of TGF-β, while maintaining receptiveness to its inducement of epithelial to mesenchymal transition (EMT) and metastatic phenotype [78].

The transcription factor serum response factor (SRF), and its binding region serum response element (SRE) in the promoters of its target genes, modulate several vital cell functions such as contractility, migration, and survival, through the enhancement of the expression of several target genes such as the oncogene c-fos. L-HDAg enhances SRF/SRE transcriptional ability [81,82].

A study that evaluated the molecular characteristics of HCC patients from Mongolia, the country with the highest incidence of HCC in the world, with an important representation of HBV–HDV coinfection, suggested a novel molecular mechanism of hepatocarcinogenesis mediated by HDV: overexpression of spectrin alpha erythrocytic 1 (SPTA1), a scaffold protein that regulates cell shape, arrangement of transmembrane proteins, and organization of organelles [56]. The mechanisms of oncogenesis are yet to be elucidated.

Lastly, HDV can promote hepatocarcinogenesis through the induction of a T-cells senescence phenotype (with upregulation of CD57) and T-cell exhaustion that cannot be fully rescued by blockage of the programmed death 1 (PD-1) pathway, which might promote intense inflammation and tissue damage, as well as an impaired immunosurveillance [83].

## 4. Clinical Features of HDV-Associated Hepatocellular Carcinoma

### 4.1. Clinical Presentation

Globally, around 8–10% of HCC cases can be attributed to HBV–HDV coinfection and an additional 30–35% to HBV mono-infection [3,4]. However, whether HBV–HDV-associated HCC is a distinct entity is yet to be determined, since few studies have focused on this subject. Also, it remains to be unraveled if geographical origin, HDV, or HBV genotypes and co-factors can modulate HBV–HDV-associated HCC clinical presentation. Lastly, the changing epidemiology and natural history of patients with HBV–HDV coinfection in the last decade, with increasing age and longer survival of these patients, as well as increasing immigration of patients from higher to lower prevalent countries, may have modulated HCC presentation.

Early studies from the 1990s suggested differences in HBV–HDV-associated HCC in Europe and Asia. An Italian study found that patients with HCC and HBV–HDV coinfection were significantly younger than HBV mono-infected patients, and frequently presented evidence of prolonged viral replication (defined as detectable serum HBV DNA and HDV RNA), prompting the hypothesis that early development of cirrhosis induced by sustained HDV active infection would boost the risk for HCC [84]. In a study from Taiwan from the 1990s, however, HDV status was not associated with age, gender, or clinical presentation of HCC. The authors considered that, differently from Italy, where HDV infection was believed to occur early as HBV–HDV coinfection, mainly through family contact or intravenous drug use [85], in Taiwan, in a non-aboriginal population, it occurred at a later age as superinfection through contact with commercial sex workers, and therefore no longer had an impact on HCC development [86]. In fact, with HBV being endemic in Taiwan, it is likely that those patients already had cirrhosis from long-standing HBV infection before HDV superinfection took place [87]. This observation is in agreement with current evidence that failed to show an impact of HDV status and replication in HCC development once cirrhosis established [26,34,36]. Furthermore, it raises the hypothesis that coinfection might carry a higher risk of HCC development compared with superinfection. However, this hypothesis has not been properly addressed in the current literature.

More recent studies support differences in the epidemiology of HBV–HDV-associated HCC in Asia and Europe, with Asian patients [88], unlike Europeans [38], presenting similar age and gender as compared with HBV mono-infection-associated HCC. However, a similar ground regardless of geographic origin is the association of coinfection with worse underlying liver disease, particularly the severity of portal hypertension and lower tumor burden [38,88].

The most relevant paper covering this topic comes from the ITA.LI.CA group [38]. In this large Italian cohort, the absolute number of HBV–HDV HCC increased around 6-fold since 2007, even though it consistently corresponded to 12–20% of HBV-associated HCC. This results from an increase in HCC prevalence, which may be the result of better care and longer survival of patients with liver cirrhosis.

HBV–HDV patients were 2 times more often female and younger by about 10 years compared to HBV mono-infected and by 20 years compared to Italian HCC patients globally. Importantly, HCC was more often diagnosed in HBV–HDV patients during surveillance, with only one fourth being diagnosed outside screening programs, as compared to up to half in HBV mono-infected patients, likely due to a particular attention given to these high-risk patients in expert centers. In accordance, the tumor burden in HBV–HDV patients was lower, presenting as smaller tumors, less frequently with a diffuse/infiltrating type, and more often within the Milan criteria. Furthermore, the underlying liver disease was more advanced in HBV–HDV coinfected patients, with worse liver function, and more frequently associated with clinically significant portal hypertension, which may have resulted in patients seeking medical attention before the onset of HCC. These differences had an impact on the management of HCC patients, with HBV–HDV coinfected patients being more often allocated to liver transplantation (LT) and less frequently to surgical resection. Even though there was only a non-significant trend to 6 months longer survival in coinfected patients, the cause of death was different, with HBV–HDV coinfected dying more often from liver failure, and HBV mono-infected from HCC progression.

Overall, these results are consistent and show that HCC commonly presents in HDV patients on the background of more advanced liver disease, while also pointing out that different routes of infection and resulting duration of active HBV and HDV replication can impact on the clinical presentation of HCC.

**Figure 1:**
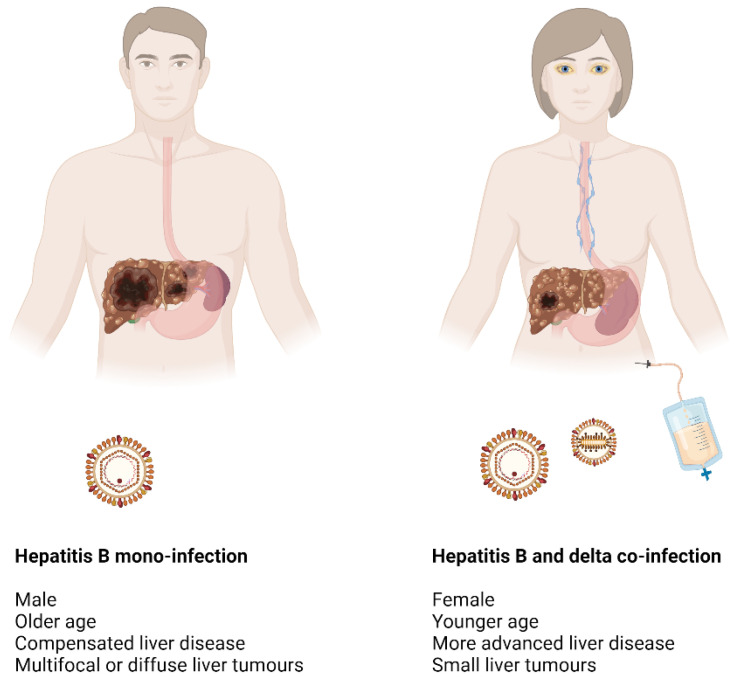
Clinical features of hepatocellular carcinoma in patients with HBV mono-infection and HDV-HBV coinfection. HBV: hepatitis B virus; HDV: hepatitis D virus. Created with BioRender.com.

The clinical features of HCC in HBV and HDV–HBV coinfected patients are summarized in Figure 1.

### 4.2. Hepatocellular Carcinoma Screening

The annual incidence of HCC in patients with HBV–HDV chronic hepatitis is estimated to be up to 1.5%, increasing to 3–4% once cirrhosis is established [18,89]. Whether antiviral treatment could hinder HCC development is still unknown. However, in a retrospective study of a mixed cirrhotic and non-cirrhotic cohort of 136 anti-HDV positive patients followed for 5 years, interferon treatment was not associated with a decreased risk of HCC development [90]. Furthermore, a retrospective study that compared 176 HBV–HDV cirrhotic patients treated with bulevirtide compared with 140 historical controls, showed that bulevirtide could not prevent HCC development, even though it decreased the risk of decompensation [91]. This study aligns with the epidemiological studies that showed that once cirrhosis has occurred, HDV RNA does not associate with HCC development [11].

The increased risk of HCC in HBV–HDV patients might warrant stricter surveillance in this population [38].

Even though non-cirrhotic HBV–HDV coinfected patients are at higher risk of HCC than HBV mono-infected patients [37], the scoring systems that were designed to select non-cirrhotic HBV patients who would benefit from a surveillance program, such as the PAGE-B [92] and the REACH-B Score [93], do not incorporate HDV coinfection. The Deltavir study, in a French cohort of 1112 HBV–HDV coinfected patients followed for a median of 3 years, suggested that the PAGE-B score could also be applied to coinfected patients, with a 5-fold increased risk of HCC development in the intermediate class (score between 10 and 17 points) and 18-fold increased risk in the elevated risk class (score equal or higher than 18 points) [20]. European and American guidelines recommend HCC surveillance when the annual risk is at least 0.2%, corresponding to the intermediate and high-risk groups in PAGE-B score for HBV mono-infected patients [94,95]. A study with a large cohort of African-born Swedish patients showed that this threshold was met at much younger ages in HDV coinfected patients (20–40 years for males and females, respectively, against 54 and 59 years in HBV), [96], suggesting that an HCC risk assessment tool for HBV–HDV patients could be a modified PAGE-D score with a different ponderation for age. The European guidelines on the management of HBV–HDV coinfection recommend HCC surveillance in patients with bridging fibrosis (METAVIR F3), irrespective of HDV RNA, with particular emphasis in the presence of other HCC risk factors such as alcohol, tobacco, metabolic factors, HCC family history, other viral co-infection such as HIV or HCV, and sub-Saharan origin given the potential exposure to aflatoxins [27].

In HBV–HDV coinfection, cirrhotic-HCC often presents with deranged liver function that may preclude therapeutic options such as surgical resection and locoregional therapy. Therefore, a greater effort should be made to diagnose tumors that still fulfill the criteria for liver transplantation. European guidelines, however, recommend an HCC screening strategy similar to other etiologies of liver cirrhosis [27].

### 4.3. Treatment Considerations

The management of HCC in a patient with HBV–HDV coinfection can be challenging. As mentioned above, the more advanced liver dysfunction can limit the therapeutic arsenal available to these patients, potentially leaving LT as the only possible option. In fact, in the ITA.LI.CA cohort, where HBV–HDV patients were younger, had less tumor burden and more deranged liver function, LT was more often performed than in HBV patients. The former derived greater benefit from LT, that was able to cure both HCC and liver disease, which is highlighted by the fact that when patients submitted to LT were excluded, HBV–HDV coinfected patients had a significantly shorter survival compared to HBV mono-infected patients (22 months vs. 36 months) [38].

HDV infection requires special considerations in the pre- and post-LT setting, and even more so in patients with HCC. As HDV requires HBV for infectivity, the strategy to prevent graft infection relies on measures directed at HBV, including nucleos(t)ise analogs (NA), which suppress viral replication, and HBIG (hepatitis B immunoglobulins), which neutralize HBsAg. The ELITA (European Liver and Intestine Transplantation Association) recommends prophylaxis with a 3rd generation NA, preferably combined with a short course (4 weeks) of HBIG in low-risk patients, i.e., those with undetectable HBV DNA pre-LT. In those with detectable HBV DNA pre-LT or HBV-related acute-on-chronic liver failure, considered high-risk, extended combined therapy is recommended, lasting for at least one year and until HBV DNA negativity is achieved [97]. HBV–HDV coinfected patients, however, are considered a special population and should receive indefinite dual prophylaxis with NA and HBIG. This recommendation stems from observations suggesting that HBsAg can rescue a latent HDV infection, a topic that has generated discord over the years [9,97]. In fact, in patients transplanted for HDV and under HBIG prophylaxis, HDAg has been shown to persist in hepatocytes for over one year [98]. This can be explained by the observation that, in addition to HBV-dependent de novo infection, HDV can also spread through cell division in the absence of HBV, establishing a non-productive infection of hepatocytes that can later be rescued by the helper virus [98,99]. However, it has not been proven that HBsAg alone, in the absence of full HBV reactivation, can efficiently restitute latent HDV infection [97].

Importantly, there are no published reports of the use of HDV-directed therapy such as bulevirtide in the post-LT setting. It seems plausible, however, that, by lowering the number of HDAg-infected hepatocytes [100], bulevirtide could play a role in preventing HBV–HDV recurrence after LT, when combined with standard prophylaxis.

Unlike HBV-associated HCC, in which nucleos(t)ide analogues or interferon-based regimens seem to decrease recurrence after surgical resection or locoregional therapies [101,102,103,104], there are no data on bulevirtide treatment in this setting.

Regarding systemic therapy, particularly in the era of immunotherapy, there is no evidence concerning the use of those therapies in the context of HBV–HDV coinfection-associated HCC. Major clinical trials did not include patients with HBV–HDV coinfection [105,106,107]. However, case reports suggest it to be safe and even to associate it with a decrease in HDV viral load, possibly as a result of enhanced anti-HDV T-cell-mediated immunity [108]. A concern could be the possibility of a lower anti-tumoral effect of immunotherapy in HBV–HDV-associated HCC since HDV is known to induce T-cell exhaustion with a blunted response to PD-1 pathway inhibition [83].

## 5. Conclusions

Since the first description of HDV by Rizzetto almost 50 years ago, the epidemiology and clinical course of HBV–HDV coinfection has shifted from young patients presenting with aggressive chronic hepatitis rapidly progressing to cirrhosis, hepatic decompensation, and death, to older patients with a more indolent course, that allows for the carcinogenic process to develop. HDV coinfection increases the risk of HBV-associated HCC, particularly through mechanisms that take place in the pre-cirrhotic stage, including enhanced carcinogenesis, but also an accelerated progression to liver cirrhosis, the main driver of HCC development. Once cirrhosis is established, the carcinogenic potential of liver damage and regenerative signal is so strong that the enhancing carcinogenic effect of HDV loses its expression and the risk of HCC development is similar between coinfected and HBV mono-infected cirrhotics.

Either because more patients with chronic HBV–HDV hepatitis, compared to HBV mono-infection hepatitis, present in the cirrhotic stage, or because those patients tend to be followed in specialized centers, the majority of coinfected HCC patients are diagnosed in surveillance programs, which is less frequent in HBV-mono-infected patients that are often diagnosed after symptoms have developed. In agreement, HCC in the setting of coinfection tends to be diagnosed in earlier stages, and those patients are more frequently offered LT. On the other hand, other therapies are more likely to be jeopardized in coinfected patients because HCC occurs in a background of severe chronic liver disease and liver failure.

With this new exciting era of HDV-directed antiviral therapy, with bulevirtide already approved and many candidate drugs in the pipeline, we can expect that a new shift in the epidemiology of HBV–HDV coinfection and associated HCC will occur in the next few years. However, if we want to impact the risk of HCC development, anti-HDV therapies probably should be started early in the course of chronic hepatitis, since after advanced fibrosis/cirrhosis has already developed, chances are it will not be successful in preventing HCC.

## Figures and Tables

**Table 1:** Main epidemiological studies evaluating the risk of HCC in HBV–HDV coinfected patients.

Author and Year	Country	Study Design	Number of Patients with HCC/HDV-HBV	Number of Patients with HCC/HBV	Main Findings
Fattovich et al., 2000 [29]	Western Europe	Retrospective	5/39	22/161	HDV increases HCC risk 3-fold; RR 3.20 [1.0–10.0].
Ji et al., 2012 [42]	Sweden	Retrospective	17/667	46/8556	Both acute and chronic HDV infections increase HCC risk; HR 3.85 [1.87–7.89] and 2.42 [1.29–4.56], respectively.
Romeo et al., 2014 [11] *	Italy	Retrospective	NA	35/193	HDV RNA levels associated with HCC in non-cirrhotics; OR 1.88 [1.11–3.19].
Kushner et al., 2015 [30]	US	Retrospective	23/1000	8/1000	HDV increases HCC risk 2-fold; aOR 2.1 [1.1–2.9].
Béguelin et al., 2017 [43]	Switzerland	Prospective	83/104	462/623	In HIV-infected patients, anti-HDV (aHR 9.30 [3.03–28.61]), and HDV RNA positivity are associated with increased HCC risk.
Brancaccio et al., 2019 [32]	Italy	Prospective	17/56	6/56	HDV increases HCC risk 2-fold in HBV suppressed patients; aHR 2.34 [0.98–6.42].
Roulot et al., 2020 [20] *	France	Retrospective	NA	72/1112	Positive HDV RNA at baseline increases HCC risk; HR 2.46 [1.35–4.48].
Wranke et al., 2023 [26]	Germany	Retrospective	20/175	12/175	Once cirrhosis is present, not HDV but the underlying cirrhosis promotes HCC development.

* Only HBV–HDV coinfected patients included. aOR: adjusted odds ratio; aHR: adjusted hazard ratio; HBV: hepatitis B virus; HDV: hepatitis delta virus; HCC: hepatocellular carcinoma; HR: hazard ratio; NA: not applicable; OR: odds ratio; RR: risk ratio.

## References

[1] Global Cancer Observatory: Cancer Today. Lyon, France: International Agency for Research on Cancer. https://gco.iarc.who.int/today.

[2] Villanueva A. (2019). Hepatocellular carcinoma. N. Engl. J. Med..

[3] Toh M.R., Wong E.Y.T., Wong S.H., Ng A.W.T., Loo L.H., Chow P.K., Ngeow J. (2023). Global epidemiology and genetics of hepatocellular carcinoma. Gastroenterology.

[4] Stockdale A.J., Kreuels B., Henrion M.Y.R., Giorgi E., Kyomuhangi I., de Martel C., Hutin Y., Geretti A.M. (2020). The global prevalence of hepatitis D virus infection: Systematic review and meta-analysis. J. Hepatol..

[5] Rizzetto M., Canese M.G., Arico S., Crivelli O., Trepo C., Bonino F., Verme G. (1977). Immunofluorescence detection of new antigen-antibody system (delta/anti-delta) associated to hepatitis B virus in liver and in serum of HBsAg carriers. Gut.

[6] Wang K.S., Choo Q.L., Weiner A.J., Ou J.H., Najarian R.C., Thayer R.M., Mullenbach G.T., Denniston K.J., Gerin J.L., Houghton M. (1986). Structure, sequence and expression of the hepatitis delta (delta) viral genome. Nature.

[7] Chen H.Y., Shen D.T., Ji D.Z., Han P.C., Zhang W.M., Ma J.F., Chen W.S., Goyal H., Pan S., Xu H.G. (2019). Prevalence and burden of hepatitis D virus infection in the global population: A systematic review and meta-analysis. Gut.

[8] Miao Z., Zhang S., Ou X., Li S., Ma Z., Wang W., Peppelenbosch M.P., Liu J., Pan Q. (2020). Estimating the global prevalence, disease progression, and clinical outcome of hepatitis delta virus infection. J. Infect. Dis..

[9] Tseligka E.D., Clement S., Negro F. (2021). HDV pathogenesis: Unravelling ariadne’s thread. Viruses.

[10] Manesis E.K., Vourli G., Dalekos G., Vasiliadis T., Manolaki N., Hounta A., Koutsounas S., Vafiadis I., Nikolopoulou G., Giannoulis G. (2013). Prevalence and clinical course of hepatitis delta infection in greece: A 13-year prospective study. J. Hepatol..

[11] Romeo R., Foglieni B., Casazza G., Spreafico M., Colombo M., Prati D. (2014). High serum levels of HDV RNA are predictors of cirrhosis and liver cancer in patients with chronic hepatitis delta. PLoS ONE.

[12] Coghill S., McNamara J., Woods M., Hajkowicz K. (2018). Epidemiology and clinical outcomes of hepatitis delta (D) virus infection in queensland, australia. Int. J. Infect. Dis..

[13] Binh M.T., Hoan N.X., Van Tong H., Giang D.P., Sy B.T., Toan N.L., Song L.H., Bang M.H., Wedemeyer H., Meyer C.G. (2018). HDV infection rates in northern vietnam. Sci. Rep..

[14] Hajarizadeh B., Grebely J., Dore G.J. (2013). Epidemiology and natural history of HCV infection. Nat. Rev. Gastroenterol. Hepatol..

[15] Lin J., Wu J.F., Zhang Q., Zhang H.W., Cao G.W. (2014). Virus-related liver cirrhosis: Molecular basis and therapeutic options. World J. Gastroenterol..

[16] Smedile A., Rizzetto M., Gerin J.L. (1994). Advances in hepatitis D virus biology and disease. Prog. Liver Dis..

[17] Rizzetto M., Verme G., Recchia S., Bonino F., Farci P., Arico S., Calzia R., Picciotto A., Colombo M., Popper H. (1983). Chronic hepatitis in carriers of hepatitis B surface antigen, with intrahepatic expression of the delta antigen. An active and progressive disease unresponsive to immunosuppressive treatment. Ann. Intern. Med..

[18] Romeo R., Del Ninno E., Rumi M., Russo A., Sangiovanni A., de Franchis R., Ronchi G., Colombo M. (2009). A 28-year study of the course of hepatitis delta infection: A risk factor for cirrhosis and hepatocellular carcinoma. Gastroenterology.

[19] Negro F., Lok A.S. (2023). Hepatitis D: A review. JAMA.

[20] Roulot D., Brichler S., Layese R., BenAbdesselam Z., Zoulim F., Thibault V., Scholtes C., Roche B., Castelnau C., Poynard T. (2020). Origin, HDV genotype and persistent viremia determine outcome and treatment response in patients with chronic hepatitis delta. J. Hepatol..

[21] Rizzetto M., Hamid S., Negro F. (2021). The changing context of hepatitis D. J. Hepatol..

[22] Kamal H., Westman G., Falconer K., Duberg A.S., Weiland O., Haverinen S., Wejstal R., Carlsson T., Kampmann C., Larsson S.B. (2020). Long-term study of hepatitis delta virus infection at secondary care centers: The impact of viremia on liver-related outcomes. Hepatology.

[23] Niro G.A., Smedile A., Ippolito A.M., Ciancio A., Fontana R., Olivero A., Valvano M.R., Abate M.L., Gioffreda D., Caviglia G.P. (2010). Outcome of chronic delta hepatitis in italy: A long-term cohort study. J. Hepatol..

[24] Buti M., Homs M., Rodriguez-Frias F., Funalleras G., Jardi R., Sauleda S., Tabernero D., Schaper M., Esteban R. (2011). Clinical outcome of acute and chronic hepatitis delta over time: A long-term follow-up study. J. Viral Hepat..

[25] Jachs M., Binter T., Schmidbauer C., Hartl L., Strasser M., Laferl H., Hametner-Schreil S., Lindorfer A., Dax K., Stauber R.E. (2021). Hepatitis D virus (HDV) prevalence in austria is low but causes considerable morbidity due to fast progression to cirrhosis. United Eur. Gastroenterol. J..

[26] Wranke A., Heidrich B., Deterding K., Hupa-Breier K.L., Kirschner J., Bremer B., Cornberg M., Wedemeyer H. (2023). Clinical long-term outcome of hepatitis D compared to hepatitis B monoinfection. Hepatol. Int..

[27] European Association for the Study of the Liver (2023). EASL clinical practice guidelines on hepatitis delta virus. J. Hepatol..

[28] Gish R.G., Wong R.J., Di Tanna G.L., Kaushik A., Kim C., Smith N.J., Kennedy P.T.F. (2024). Association of hepatitis delta virus with liver morbidity and mortality: A systematic literature review and meta-analysis. Hepatology.

[29] Fattovich G., Giustina G., Christensen E., Pantalena M., Zagni I., Realdi G., Schalm S.W. (2000). Influence of hepatitis delta virus infection on morbidity and mortality in compensated cirrhosis type B. The european concerted action on viral hepatitis (eurohep). Gut.

[30] Kushner T., Serper M., Kaplan D.E. (2015). Delta hepatitis within the veterans affairs medical system in the united states: Prevalence, risk factors, and outcomes. J. Hepatol..

[31] Bockmann J.H., Grube M., Hamed V., von Felden J., Landahl J., Wehmeyer M., Giersch K., Hall M.T., Murray J.M., Dandri M. (2020). High rates of cirrhosis and severe clinical events in patients with HBV/HDV co-infection: Longitudinal analysis of a german cohort. BMC Gastroenterol..

[32] Brancaccio G., Fasano M., Grossi A., Santantonio T.A., Gaeta G.B. (2019). Clinical outcomes in patients with hepatitis D, cirrhosis and persistent hepatitis B virus replication, and receiving long-term tenofovir or entecavir. Aliment. Pharmacol. Ther..

[33] Cross T.J., Rizzi P., Horner M., Jolly A., Hussain M.J., Smith H.M., Vergani D., Harrison P.M. (2008). The increasing prevalence of hepatitis delta virus (HDV) infection in south london. J. Med. Virol..

[34] Alfaiate D., Clement S., Gomes D., Goossens N., Negro F. (2020). Chronic hepatitis D and hepatocellular carcinoma: A systematic review and meta-analysis of observational studies. J. Hepatol..

[35] Chang T.E., Su C.W., Huang Y.S., Huang Y.H., Hou M.C., Wu J.C. (2022). Hepatitis D virus dual infection increased the risk of hepatocellular carcinoma compared with hepatitis B virus mono infection: A meta-analysis. J. Chin. Med. Assoc..

[36] Kamal H., Fornes R., Simin J., Stal P., Duberg A.S., Brusselaers N., Aleman S. (2021). Risk of hepatocellular carcinoma in hepatitis B and D virus co-infected patients: A systematic review and meta-analysis of longitudinal studies. J. Viral Hepat..

[37] Amakye D., Forlemu R., Kikelomo O., Soladoye E., Moparty H., Forlemu A., Bandaru P., Lohani S., Kavuri S., Garzon-Siatoya W. (2024). Hepatitis B versus hepatitis B delta co-infection and risk of hepatocellular carcinoma in non-cirrhotic liver. Gastroenterology.

[38] Giannini E.G., Pasta A., Pieri G., Plaz Torres M.C., Marseglia M., Pelizzaro F., Sangiovanni A., Cabibbo G., Ghittoni G., Di Marco M. (2024). Characteristics and outcome of anti-hepatitis D virus positive patients with hepatocellular carcinoma. Liver Int..

[39] Bonacini M. (2024). Delta virus infection and hepatocellular carcinoma. Liver Int..

[40] Brancaccio G., Coco B., Nardi A., Quaranta M.G., Tosti M.E., Ferrigno L., Cacciola I., Messina V., Chessa L., Morisco F. (2023). Trends in chronic hepatitis B virus infection in italy over a 10-year period: Clues from the nationwide piter and master cohorts toward elimination. Int. J. Infect. Dis..

[41] Wedemeyer H., Leus M., Battersby T.R., Glenn J., Gordien E., Kamili S., Kapoor H., Kessler H.H., Lenz O., Lutgehetmann M. (2023). HDV RNA assays: Performance characteristics, clinical utility, and challenges. Hepatology.

[42] Ji J., Sundquist K., Sundquist J. (2012). A population-based study of hepatitis D virus as potential risk factor for hepatocellular carcinoma. J. Natl. Cancer Inst..

[43] Beguelin C., Moradpour D., Sahli R., Suter-Riniker F., Luthi A., Cavassini M., Gunthard H.F., Battegay M., Bernasconi E., Schmid P. (2017). Hepatitis delta-associated mortality in hiv/hbv-coinfected patients. J. Hepatol..

[44] Oyunsuren T., Kurbanov F., Tanaka Y., Elkady A., Sanduijav R., Khajidsuren O., Dagvadorj B., Mizokami M. (2006). High frequency of hepatocellular carcinoma in mongolia; association with mono-, or co-infection with hepatitis C, B, and delta viruses. J. Med. Virol..

[45] Desai A., Sandhu S., Lai J.P., Sandhu D.S. (2019). Hepatocellular carcinoma in non-cirrhotic liver: A comprehensive review. World J. Hepatol..

[46] Choi S.H., Jeong S.H., Hwang S.B. (2007). Large hepatitis delta antigen modulates transforming growth factor-beta signaling cascades: Implication of hepatitis delta virus-induced liver fibrosis. Gastroenterology.

[47] Park C.Y., Oh S.H., Kang S.M., Lim Y.S., Hwang S.B. (2009). Hepatitis delta virus large antigen sensitizes to TNF-alpha-induced NF-kappaB signaling. Mol. Cells.

[48] Giersch K., Allweiss L., Volz T., Helbig M., Bierwolf J., Lohse A.W., Pollok J.M., Petersen J., Dandri M., Lutgehetmann M. (2015). Hepatitis delta co-infection in humanized mice leads to pronounced induction of innate immune responses in comparison to hbv mono-infection. J. Hepatol..

[49] Ringelhan M., O’Connor T., Protzer U., Heikenwalder M. (2015). The direct and indirect roles of HBV in liver cancer: Prospective markers for HCC screening and potential therapeutic targets. J. Pathol..

[50] Chen C.J., Yang H.I., Su J., Jen C.L., You S.L., Lu S.N., Huang G.T., Iloeje U.H., Group R.-H.S. (2006). Risk of hepatocellular carcinoma across a biological gradient of serum hepatitis B virus DNA level. JAMA.

[51] Alfaiate D., Lucifora J., Abeywickrama-Samarakoon N., Michelet M., Testoni B., Cortay J.C., Sureau C., Zoulim F., Deny P., Durantel D. (2016). HDV RNA replication is associated with HBV repression and interferon-stimulated genes induction in super-infected hepatocytes. Antivir. Res..

[52] Liaw Y.F., Dong J.T., Chiu K.W., Sheen I.S., Chu C.M. (1991). Why most patients with hepatitis delta virus infection are seronegative for hepatitis B e antigen. A prospective controlled study. J. Hepatol..

[53] Padarath K., Deroubaix A., Kramvis A. (2023). The complex role of HBeAg and its precursors in the pathway to hepatocellular carcinoma. Viruses.

[54] Diaz G., Engle R.E., Tice A., Melis M., Montenegro S., Rodriguez-Canales J., Hanson J., Emmert-Buck M.R., Bock K.W., Moore I.N. (2018). Molecular signature and mechanisms of hepatitis D virus-associated hepatocellular carcinoma. Mol. Cancer Res..

[55] Yu Z., Ma X., Zhang W., Chang X., An L., Niu M., Chen Y., Sun C., Yang Y. (2021). Microarray data mining and preliminary bioinformatics analysis of hepatitis D virus-associated hepatocellular carcinoma. BioMed Res. Int..

[56] Candia J., Bayarsaikhan E., Tandon M., Budhu A., Forgues M., Tovuu L.O., Tudev U., Lack J., Chao A., Chinburen J. (2020). The genomic landscape of mongolian hepatocellular carcinoma. Nat. Commun..

[57] Puigvehi M., Moctezuma-Velazquez C., Villanueva A., Llovet J.M. (2019). The oncogenic role of hepatitis delta virus in hepatocellular carcinoma. JHEP Rep..

[58] Guilhot S., Huang S.N., Xia Y.P., La Monica N., Lai M.M., Chisari F.V. (1994). Expression of the hepatitis delta virus large and small antigens in transgenic mice. J. Virol..

[59] Williams V., Brichler S., Khan E., Chami M., Deny P., Kremsdorf D., Gordien E. (2012). Large hepatitis delta antigen activates stat-3 and NF-kappaB via oxidative stress. J. Viral Hepat..

[60] Chen M., Du D., Zheng W., Liao M., Zhang L., Liang G., Gong M. (2019). Small hepatitis delta antigen selectively binds to target mrna in hepatic cells: A potential mechanism by which hepatitis d virus downregulates glutathione s-transferase p1 and induces liver injury and hepatocarcinogenesis. Biochem. Cell Biol..

[61] Liu M., Cui L.H., Li C.C., Zhang L. (2015). Association of APC, GSTP1 and SOCS1 promoter methylation with the risk of hepatocellular carcinoma: A meta-analysis. Eur. J. Cancer Prev..

[62] Li Y., Cai Y., Chen H., Mao L. (2018). Clinical significance and association of GSTP1 hypermethylation with hepatocellular carcinoma: A meta-analysis. J. Cancer Res. Ther..

[63] Chen Y.L., Tseng H.S., Kuo W.H., Yang S.F., Chen D.R., Tsai H.T. (2010). Glutathione s-transferase p1 (GSTP1) gene polymorphism increases age-related susceptibility to hepatocellular carcinoma. BMC Med. Genet..

[64] Wang Z., Qu K., Niu W., Lin T., Xu X., Huang Z., Liu S., Liu S., Chang H., Liu Y. (2016). Glutathione s-transferase p1 gene rs4147581 polymorphism predicts overall survival of patients with hepatocellular carcinoma: Evidence from an enlarged study. Tumour Biol..

[65] Gu Y., Mohammad I.S., Liu Z. (2020). Overview of the STAT-3 signaling pathway in cancer and the development of specific inhibitors. Oncol. Lett..

[66] Benegiamo G., Vinciguerra M., Guarnieri V., Niro G.A., Andriulli A., Pazienza V. (2013). Hepatitis delta virus induces specific DNA methylation processes in huh-7 liver cancer cells. FEBS Lett..

[67] Nagaraju G.P., Dariya B., Kasa P., Peela S., El-Rayes B.F. (2022). Epigenetics in hepatocellular carcinoma. Semin. Cancer Biol..

[68] Denechaud P.D., Fajas L., Giralt A. (2017). E2f1, a novel regulator of metabolism. Front. Endocrinol..

[69] Gupta R., Kadhim M.M., Turki Jalil A., Obayes A.M., Aminov Z., Alsaikhan F., Ramirez-Coronel A.A., Ramaiah P., Tayyib N.A., Luo X. (2023). Multifaceted role of NF-kappaB in hepatocellular carcinoma therapy: Molecular landscape, therapeutic compounds and nanomaterial approaches. Environ. Res..

[70] Mendes M., Perez-Hernandez D., Vazquez J., Coelho A.V., Cunha C. (2013). Proteomic changes in hek-293 cells induced by hepatitis delta virus replication. J. Proteom..

[71] Tavanez J.P., Caetano R., Branco C., Brito I.M., Miragaia-Pereira A., Vassilevskaia T., Quina A.S., Cunha C. (2020). Hepatitis delta virus interacts with splicing factor SF3B155 and alters pre-mRNA splicing of cell cycle control genes. FEBS J..

[72] Mu J.Y., Tian J.X., Chen Y.J. (2021). LncRNA RBM5-AS1 promotes cell proliferation and invasion by epigenetically silencing mir-132/212 in hepatocellular carcinoma cells. Cell Biol. Int..

[73] Liao F.T., Lee Y.J., Ko J.L., Tsai C.C., Tseng C.J., Sheu G.T. (2009). Hepatitis delta virus epigenetically enhances clusterin expression via histone acetylation in human hepatocellular carcinoma cells. J. Gen. Virol..

[74] Kang Y.K., Hong S.W., Lee H., Kim W.H. (2004). Overexpression of clusterin in human hepatocellular carcinoma. Hum. Pathol..

[75] Xiu P., Dong X.F., Li X.P., Li J. (2015). Clusterin: Review of research progress and looking ahead to direction in hepatocellular carcinoma. World J. Gastroenterol..

[76] Lau S.H., Sham J.S., Xie D., Tzang C.H., Tang D., Ma N., Hu L., Wang Y., Wen J.M., Xiao G. (2006). Clusterin plays an important role in hepatocellular carcinoma metastasis. Oncogene.

[77] Zhang Q., Matsuura K., Kleiner D.E., Zamboni F., Alter H.J., Farci P. (2016). Analysis of long noncoding RNA expression in hepatocellular carcinoma of different viral etiology. J. Transl. Med..

[78] Gonzalez-Sanchez E., Vaquero J., Fernandez-Barrena M.G., Lasarte J.J., Avila M.A., Sarobe P., Reig M., Calvo M., Fabregat I. (2021). The tgf-beta pathway: A pharmacological target in hepatocellular carcinoma?. Cancers.

[79] Guo Y., Zhu J., Xu X., Shen B., Shen Z., Li B., Li F., Gu T., Cai X., Dong H. (2022). Tgf-beta/yb-1/atg7 axis promotes the proliferation of hepatic progenitor cells and liver fibrogenesis. Biochim. Biophys. Acta Mol. Basis Dis..

[80] Wu K., Ding J., Chen C., Sun W., Ning B.F., Wen W., Huang L., Han T., Yang W., Wang C. (2012). Hepatic transforming growth factor beta gives rise to tumor-initiating cells and promotes liver cancer development. Hepatology.

[81] Goto T., Kato N., Ono-Nita S.K., Yoshida H., Otsuka M., Shiratori Y., Omata M. (2000). Large isoform of hepatitis delta antigen activates serum response factor-associated transcription. J. Biol. Chem..

[82] Goto T., Kato N., Yoshida H., Otsuka M., Moriyama M., Shiratori Y., Koike K., Matsumura M., Omata M. (2003). Synergistic activation of the serum response element-dependent pathway by hepatitis B virus X protein and large-isoform hepatitis delta antigen. J. Infect. Dis..

[83] Schirdewahn T., Grabowski J., Owusu Sekyere S., Bremer B., Wranke A., Lunemann S., Schlaphoff V., Kirschner J., Hardtke S., Manns M.P. (2017). The third signal cytokine interleukin 12 rather than immune checkpoint inhibitors contributes to the functional restoration of hepatitis d virus-specific t cells. J. Infect. Dis..

[84] Verme G., Brunetto M.R., Oliveri F., Baldi M., Forzani B., Piantino P., Ponzetto A., Bonino F. (1991). Role of hepatitis delta virus infection in hepatocellular carcinoma. Dig. Dis. Sci..

[85] Stroffolini T., Ferrigno L., Cialdea L., Catapano R., Palumbo F., Novaco F., Moiraghi A., Galanti C., Bernacchia R., Mele A. (1994). Incidence and risk factors of acute delta hepatitis in italy: Results from a national surveillance system. Seieva collaborating group. J. Hepatol..

[86] Huo T.I., Wu J.C., Lai C.R., Lu C.L., Sheng W.Y., Lee S.D. (1996). Comparison of clinico-pathological features in hepatitis B virus-associated hepatocellular carcinoma with or without hepatitis D virus superinfection. J. Hepatol..

[87] Huo T.I., Wu J.C., Lin R.Y., Sheng W.Y., Chang F.Y., Lee S.D. (1997). Decreasing hepatitis D virus infection in taiwan: An analysis of contributory factors. J. Gastroenterol. Hepatol..

[88] Abbas Z., Qureshi M., Hamid S., Jafri W. (2012). Hepatocellular carcinoma in hepatitis D: Does it differ from hepatitis B monoinfection?. Saudi J. Gastroenterol..

[89] Jang T.Y., Wei Y.J., Liu T.W., Yeh M.L., Liu S.F., Hsu C.T., Hsu P.Y., Lin Y.H., Liang P.C., Hsieh M.H. (2021). Role of hepatitis D virus infection in development of hepatocellular carcinoma among chronic hepatitis B patients treated with nucleotide/nucleoside analogues. Sci. Rep..

[90] Wranke A., Serrano B.C., Heidrich B., Kirschner J., Bremer B., Lehmann P., Hardtke S., Deterding K., Port K., Westphal M. (2017). Antiviral treatment and liver-related complications in hepatitis delta. Hepatology.

[91] Degasperi E., Silvestri A., Anolli M.P., Sambarino D., Borghi M., Perbellini M., Facchetti F., Soffredini R., Monico S., de Lédinghen V. (2024). Bulevirtide monotherapy prevents liver decompensation and reduces mortality in patients with HDV-related cirrhosis: A case control study with propensity score weighted analysis. J. Hepatol..

[92] Papatheodoridis G., Dalekos G., Sypsa V., Yurdaydin C., Buti M., Goulis J., Calleja J.L., Chi H., Manolakopoulos S., Mangia G. (2016). Page-B predicts the risk of developing hepatocellular carcinoma in caucasians with chronic hepatitis B on 5-year antiviral therapy. J. Hepatol..

[93] Yang H.I., Yuen M.F., Chan H.L., Han K.H., Chen P.J., Kim D.Y., Ahn S.H., Chen C.J., Wong V.W., Seto W.K. (2011). Risk estimation for hepatocellular carcinoma in chronic hepatitis B (REACH-B): Development and validation of a predictive score. Lancet Oncol..

[94] Singal A.G., Llovet J.M., Yarchoan M., Mehta N., Heimbach J.K., Dawson L.A., Jou J.H., Kulik L.M., Agopian V.G., Marrero J.A. (2023). AASLD practice guidance on prevention, diagnosis, and treatment of hepatocellular carcinoma. Hepatology.

[95] European Association for the Study of the Liver (2018). EASL clinical practice guidelines: Management of hepatocellular carcinoma. J. Hepatol..

[96] Kamal H., Ingre M., Stal P., Westman G., Bruce D., Wedemeyer H., Duberg A.S., Aleman S. (2023). Age-specific and sex-specific risks for HCC in african-born persons with chronic hepatitis B without cirrhosis. Hepatol. Commun..

[97] Duvoux C., Belli L.S., Fung J., Angelico M., Buti M., Coilly A., Cortesi P., Durand F., Feray C., Fondevila C. (2021). 2020 position statement and recommendations of the european liver and intestine transplantation association (ELITA): Management of hepatitis B virus-related infection before and after liver transplantation. Aliment. Pharmacol. Ther..

[98] Zhang Z., Ni Y., Lempp F.A., Walter L., Mutz P., Bartenschlager R., Urban S. (2022). Hepatitis D virus-induced interferon response and administered interferons control cell division-mediated virus spread. J. Hepatol..

[99] Giersch K., Helbig M., Volz T., Allweiss L., Mancke L.V., Lohse A.W., Polywka S., Pollok J.M., Petersen J., Taylor J. (2014). Persistent hepatitis D virus mono-infection in humanized mice is efficiently converted by hepatitis B virus to a productive co-infection. J. Hepatol..

[100] Allweiss L., Volmari A., Suri V., Wallin J.J., Flaherty J.F., Manuilov D., Downie B., Lutgehetmann M., Bockmann J.H., Urban S. (2024). Blocking viral entry with bulevirtide reduces the number of HDV-infected hepatocytes in human liver biopsies. J. Hepatol..

[101] Wu C.Y., Chen Y.J., Ho H.J., Hsu Y.C., Kuo K.N., Wu M.S., Lin J.T. (2012). Association between nucleoside analogues and risk of hepatitis B virus-related hepatocellular carcinoma recurrence following liver resection. JAMA.

[102] Lee T.Y., Lin J.T., Zeng Y.S., Chen Y.J., Wu M.S., Wu C.Y. (2016). Association between nucleos(t)ide analog and tumor recurrence in hepatitis B virus-related hepatocellular carcinoma after radiofrequency ablation. Hepatology.

[103] Luo J.X., Zhang Y., Hu X.Y., Xiang N. (2024). Interferon therapy improves survival in patients with hepatitis B virus-related hepatocellular carcinoma after curative surgery: A meta-analysis. Hepatol. Int..

[104] Chen X.X., Cheng J.W., Huang A., Zhang X., Wang J., Fan J., Zhou J., Yang X.R. (2017). The effect of antiviral therapy on patients with hepatitis B virus-related hepatocellular carcinoma after curative resection: A systematic review and meta-analysis. Oncol. Targets Ther..

[105] Finn R.S., Qin S., Ikeda M., Galle P.R., Ducreux M., Kim T.Y., Kudo M., Breder V., Merle P., Kaseb A.O. (2020). Atezolizumab plus bevacizumab in unresectable hepatocellular carcinoma. N. Engl. J. Med..

[106] Abou-Alfa G.K., Lau G., Kudo M., Chan S.L., Kelley R.K., Furuse J., Sukeepaisarnjaroen W., Kang Y.K., Van Dao T., De Toni E.N. (2022). Tremelimumab plus durvalumab in unresectable hepatocellular carcinoma. NEJM Evid..

[107] Ren Z., Xu J., Bai Y., Xu A., Cang S., Du C., Li Q., Lu Y., Chen Y., Guo Y. (2021). Sintilimab plus a bevacizumab biosimilar (ibi305) versus sorafenib in unresectable hepatocellular carcinoma (orient-32): A randomised, open-label, phase 2–3 study. Lancet Oncol..

[108] Jachs M., Scheiner B., Pinter M. (2022). Immunotherapy for hepatocellular carcinoma in a patient with hepatitis B virus and hepatitis delta virus coinfection. J. Hepatol..

